# Study of instability mechanisms of trucks turning right at long downhill T-junctions based on Trucksim simulation

**DOI:** 10.1371/journal.pone.0282779

**Published:** 2023-03-08

**Authors:** Shibo Zhang, Yishan Liu, Pingfei Li, Li Wang, Xixi Zhou, Yi Li, Yong Luo

**Affiliations:** 1 School of Automobile and Transportation, Xihua University, Chengdu, China; 2 Sichuan Xihua Jiaotong Forensic Center, Chengdu, China; 3 Traffic Police Headquarters of Sichuan Public Security Department, Chengdu, China; 4 Sichuan Vocational and Technical College of Communications, Chengdu, China; Southwest Jiaotong University, CHINA

## Abstract

The aim of this study was to investigate the influence of road factors on the safety speed threshold of a lorry turning right around a corner at a the bottom of a long downhill T-junction. Trucksim simulation software was chosen to construct a model for investigating the turning instability mechanism. A three-axle truck was chosen as the simulation vehicle and road adhesion coefficients of 0.2–0.75, road super-elevations of -2–8%, turning radii of 20–100 m, and vehicle overcharge of 0–100% selected for tuning. Simulation experiments were carried out for different bending conditions, investigating the effects of each influencing factor on the destabilization speed threshold using the control variable method. The vehicle’s lateral load transfer rate and lateral acceleration were indicators for determining whether a truck was unstable. The results showed that: a) the turning radius had the most significant influence on the speed threshold for cornering instability; b) the road surface adhesion coefficient and vehicle overweight had secondary effects; and c) the road height had a general influence.

## Introduction

A T-junction at the bottom of a slope on a long downhill section is a poor way to cross a road. Because the relevant road facilities are missing or not properly set up, downhill lorries are prone to destabilization rollovers or skidding, overturning small vehicles or pedestrians and possibly resulting in casualties and property damage [1]. The long downhill T-crossing is recommended in the relevant engineering standards [2]. However, the reason why long downhill T-junctions with safety hazards are not uncommon is due to topography, land use, funding, history, operation and management, maintenance, and other practical reasons. The mismatch between the cross slope and right turn curve and the large step difference between the safe speed of vehicles turning right and the running speed of the upstream mainline are characteristics of a right turn road at a long downhill T-junction. At the moment, trucks account for a high proportion of vehicles on roads in China [3], and the truck itself has poor operational performance, complex overall structure, and other characteristics, such that truck accidents are frequently more severe than other events [4]. Therefore, to improve the safety of truck traffic, a more profound understanding of the destabilization mechanism of trucks turning right at long downhill T-junctions is required.

The existing research on truck traffic safety is mainly focused on two aspects: turning [5, 6] and the downhill [7–9]. Wan et al. have investigated the influence of driving behavior on the driving characteristics of vehicles through simulation experiments on long downhill road segments and optimized vehicle gear selection decisions [7]. Li et al. have investigated the maximum longitudinal slope length for stable operation of freight wagons through simulation [8]. Pan et al. have established a vehicle model on the Trucksim platform and discussed the influence of different factors on the critical value of the safe body through simulation experiments of multiple ensembles with different corner radii and different center of gravity heights but did not discuss the influence of the placement of road arches at road corners [10]. Jiao Zhuobin has performed a comparative study on the different influencing factors of the speed of safety vehicles at turns by simulating different road environments, but discussion of the combination of long turns has been lacking [11]. Tang et al. have performed orthogonal simulation experiments on different curve radii and vehicle velocities and established a mathematical response surface model of the curve radii and safe velocities of a vehicle [12]. Andrew Tarko et al. have considered the complex path and rollover experienced by semitrailers and other trucks on circular drives and proposed a model for assessing the tendency of trucks to rollover on existing circular intersections and other curves [13].

In research on vehicle instability in sharp turns or sections of hillslopes, the focus of this study was on the impact of road environmental factors, such as turn radius, superelevation, and road adhesion coefficient [11], on safe speed and the relationship between vehicle characteristics and safe speed. In the TTR [10]vehicle rollover prediction method, by Lusetti B [14], the lateral load transfer rate method (*P*_*LTRd*_) [12], and other methods have been primarily used to judge whether a truck has a rollover and sideslip [15]. Furthermore, research on T-shaped long downhill intersections turning right has been limited [16–18]. At the same time, considering the characteristics of a long downhill T-intersection right-turn road, the influencing factors and mechanisms causing truck right-turn instability were studied and, on this basis, suggestions made for the setting of road facilities and vehicle driving.

## Model

### Long downhill T-junction

**Fig 1:**
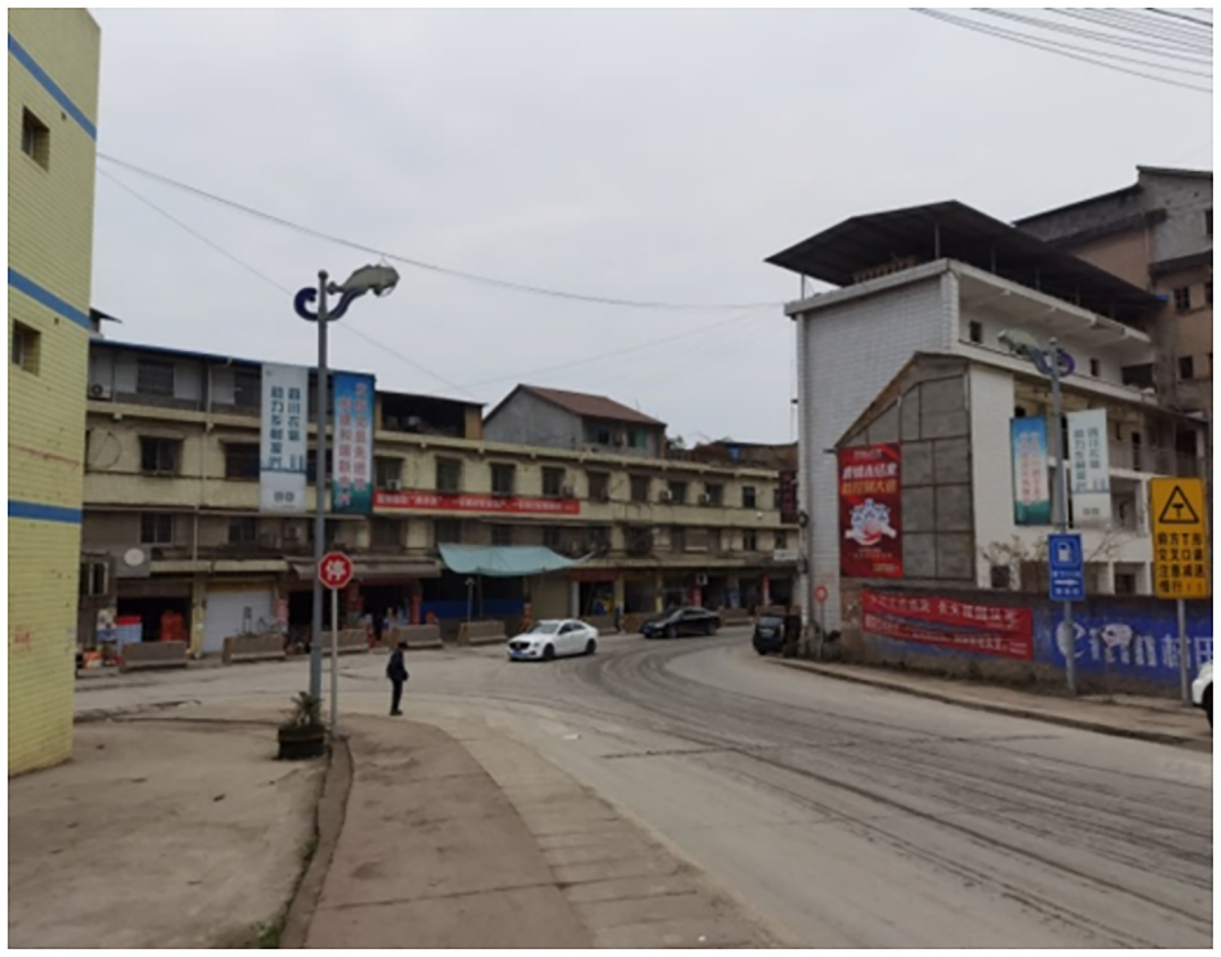
A long downhill T-junction in Zizhong, Sichuan.

**Fig 2:**
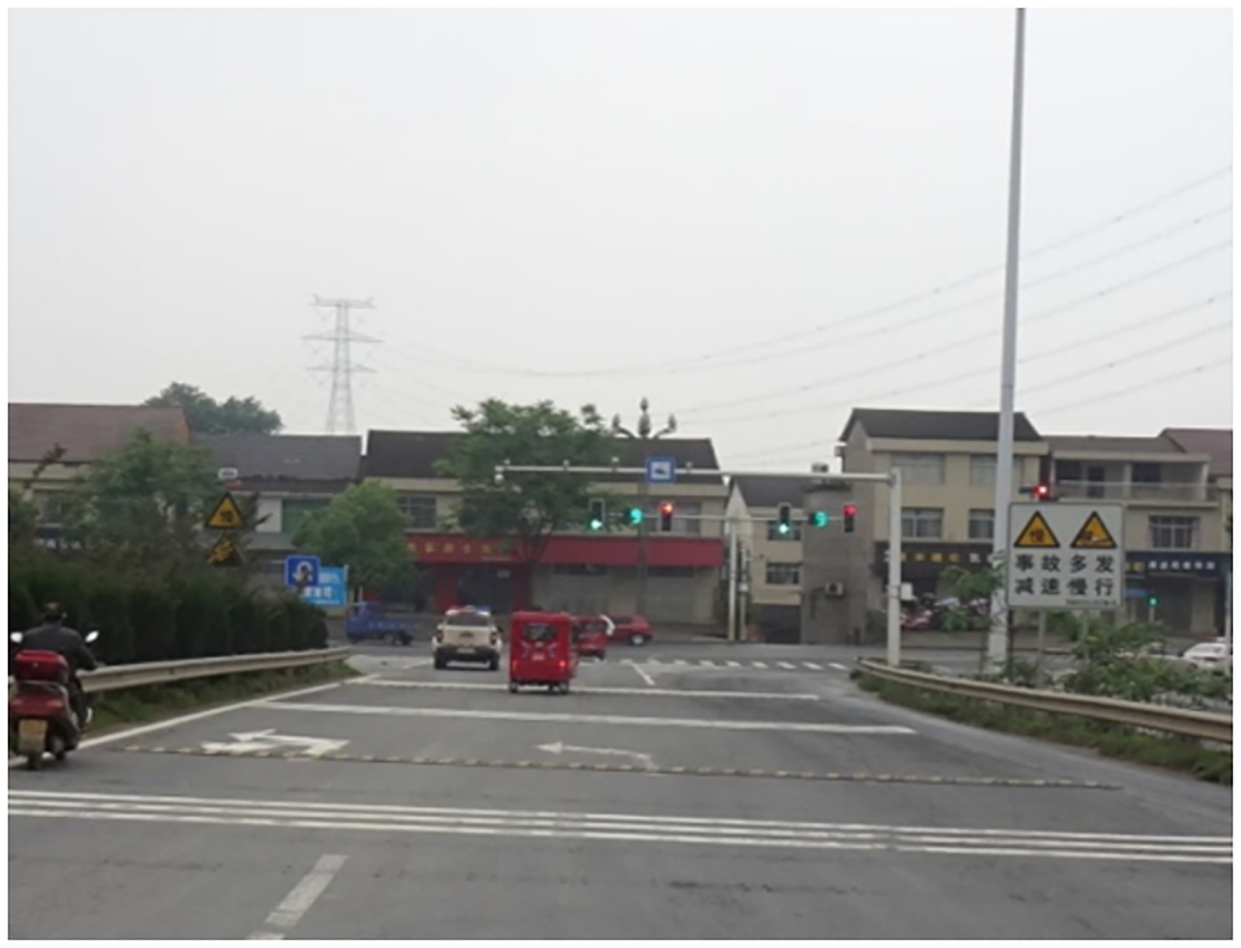
A long downhill T-junction in Changde, Hunan.

Truck accidents occur frequently on ramps and curves. As a combination of the two, a long downhill T-junction has the characteristics of a special structure, strict safety driving requirements, and extremely high construction quality requirements. Although Chinese existing road specifications have provisions for ramps, curves, and design speeds, there are many uncertainties when the two are combined, such that, for example, the vehicle instability speed threshold is unknown. In addition, when heavy-duty trucks are running on long downhill sections, brake efficiency can easily decrease or fail due to temperature increases caused by long-term braking. This will lead to truck instability if the initial speed is too high when the truck enters the slope bend. Long downhill T-junctions in Zizhong, Sichuan, and Changde, Hunan, are shown in Figs 1 and 2, respectively. Both are at the intersection of an expressway connecting line and a national road. And truck instability accidents often occur in the right-turning direction of the downhill, which can cause death in severe cases.

### Road model

**Fig 3:**
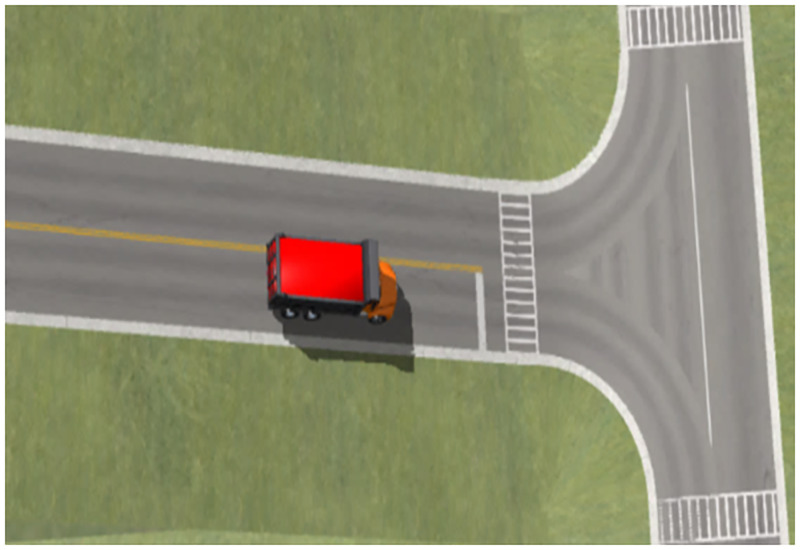
Simulation model of typical scene of right turn at a long downhill T-junction.

The road scene is a T-shaped intersection between the bottom of a long downhill road and another road. The construction of a road model mainly considers two aspects: road geometry and pavement conditions. The plane alignment of the right-turning road used here was a combination of a straight line + circular curve + straight line. The radius of the circular curve, right-turning transverse slope (superelevation), and road adhesion coefficient were variable parameters and were used according to experimental needs. A typical scene simulation model of a long downhill T-junction right turn based on Trucksim is shown in Fig 3.

### Truck model

**Fig 4:**
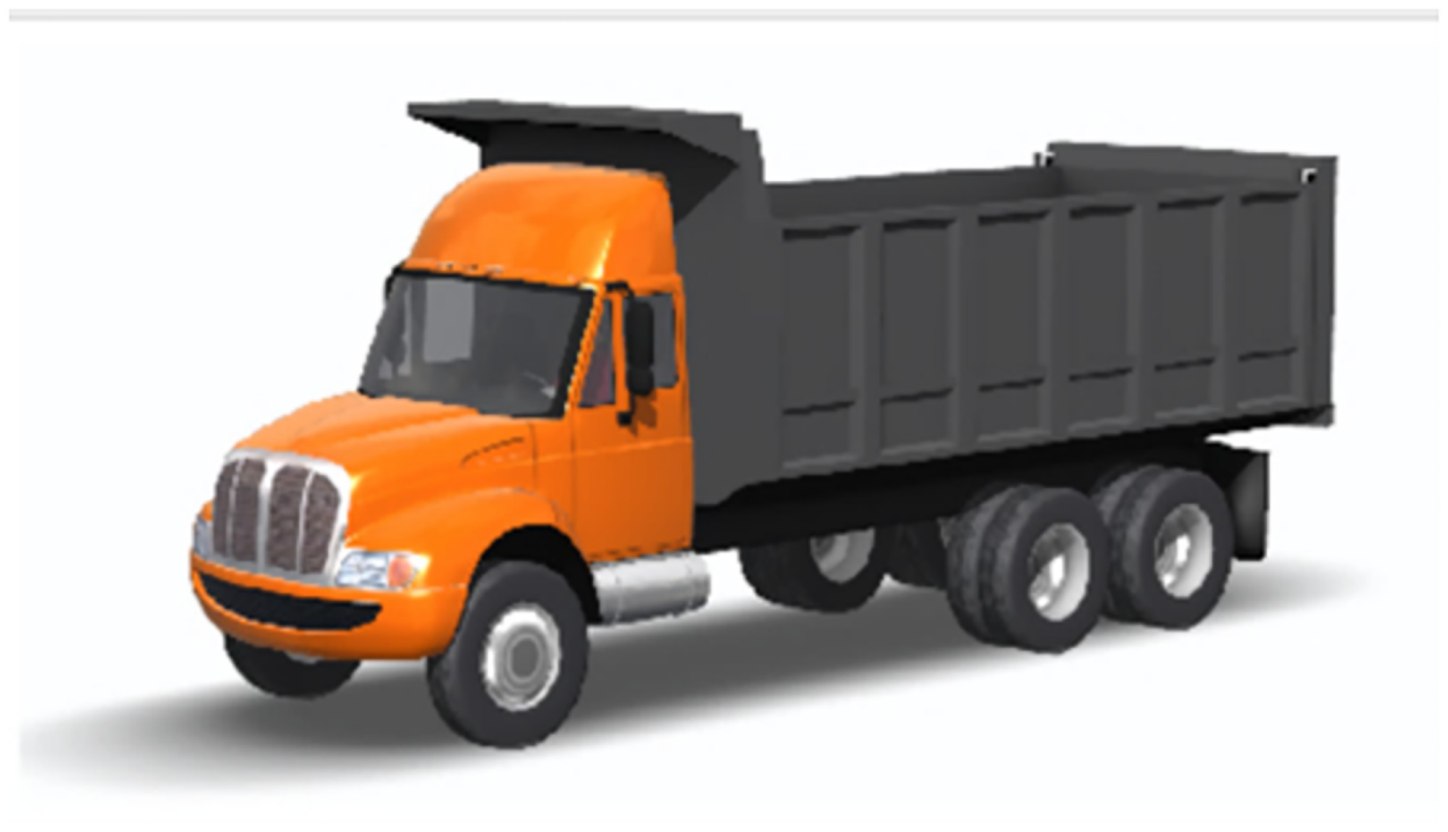
Simulation experimental vehicle.

To ensure the validity of the simulation model, a domestic truck was used as the simulation prototype. The vehicle dynamics simulation software (Trucksim) was used to establish a three-axis truck simulation model and the simulation parameters were measured according to the actual vehicle; other data were set to default. The main parameters are shown in Table 1 and vehicle model in Fig 4.

**Table 1:** Experimental vehicle parameters.

Name	Value
Curb Weight (kg)	12500
Roll Inertia (kg/m^2^)	6879
Wheelbase (mm)	4050/1350
Axle Track (mm)	2040/1880/1880
Engine Maximum Power (kW)	330
All Dimensions (mm)	8350 × 3600 × 2500

### Driving model

Driving is a very complicated behavior. It includes the driver’s ability level and safety awareness in emergency situations. The driving mode has a significant impact on the vehicle and the surrounding environment, which is related to the safety of driving. Driving behaviors such as acceleration, deceleration, steering, and shifting, have a direct impact on truck cornering stability. However, the immediate factors that determine whether the truck is unstable include the transient conditions of the truck, such as speed, turning radius, and load. These conditions are the result of driving behavior and, thus, the driver closed-loop control mode was selected here. The simulation vehicle could adjust the driving strategy according to the actual situation of the road section and the vehicle status, but here the vehicle would not accelerate, decelerate, brake, or shift during the turn.

## Analysis

### Mechanical model analysis

**Fig 5:**
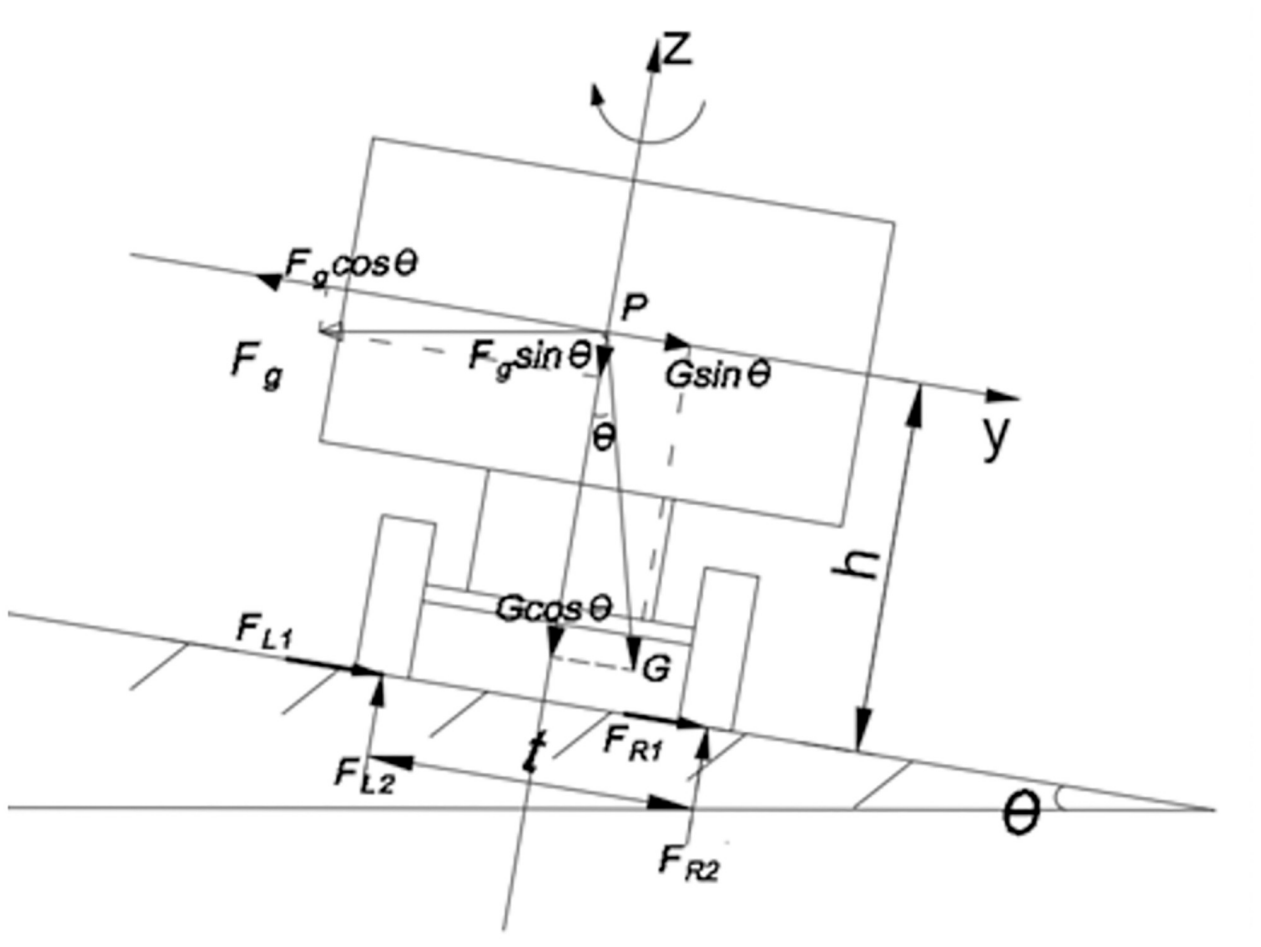
Force model of the vehicle. Note: *F*_*L*1_,*F*_*R*1_ were left-hand wheel side forces, N; *F*_*L*2_,*F*_*R*2_ right-hand wheel side forces, N; *G* the vehicle gravitation, N; *F*_*g*_ the centrifugal force exerted on the vehicle, N; *θ* the cross-slope angle of the road, °; *P* the vehicle center of mass; *h* the height of the vehicle centroid, m; and *t* the axle track, m.

A simplified model of vehicle steering dynamics was established to analyze the force condition of vehicle steering and its influence on driving stability(Fig 5). Vehicles running on curves are subjected to centrifugal force (*F*_*g*_), ground support (*F*_*L*2_,*F*_*R*2_) and lateral adhesion (*F*_*L*1_,*F*_*R*1_) and these three forces together affect vehicle driving stability [19]. To simplify the vehicle model, the elastic deformation of the suspension and tires of the vehicle were ignored when analyzing truck forces. The truck in this study was assumed to be rigid.

### Sideslip condition index

If the turning speed of the vehicle is too large near the grip limit, most of the centrifugal force is lost from the grip, and the sidewall can only offer a very small grip. Too much lateral acceleration causes the vehicle to slip. From Newton’s second law, one can derive the equilibrium equation, expressed in Eq (1):

$$\begin{array}{c} F_{c}=ma_{y}=\mu mg=F_{g}\;\text{cos}\;\theta -G\;\text{sin}\;\theta \end{array}$$ (1)

Note:where *F*_*c*_ the lateral force, *m* the truck weight, *a*_*y*_ the transverse acceleration, and *μ* the road adhesion coefficient.

From this, the adhesion provided by the road surface to the vehicle can balance the lateral centrifugal force on the vehicle. Adhesion coefficients to the road surface can be appropriately adjusted to reduce the risk of vehicle sideslip. While controlling the lateral acceleration of the vehicle bending to reduce sideslip, the lateral acceleration (*a*_*y*_) due to bending by heavy vehicles should not be greater than the threshold value of 0.3 *g* or sideslip will occur [20].

### State flip index

**Fig 6:**
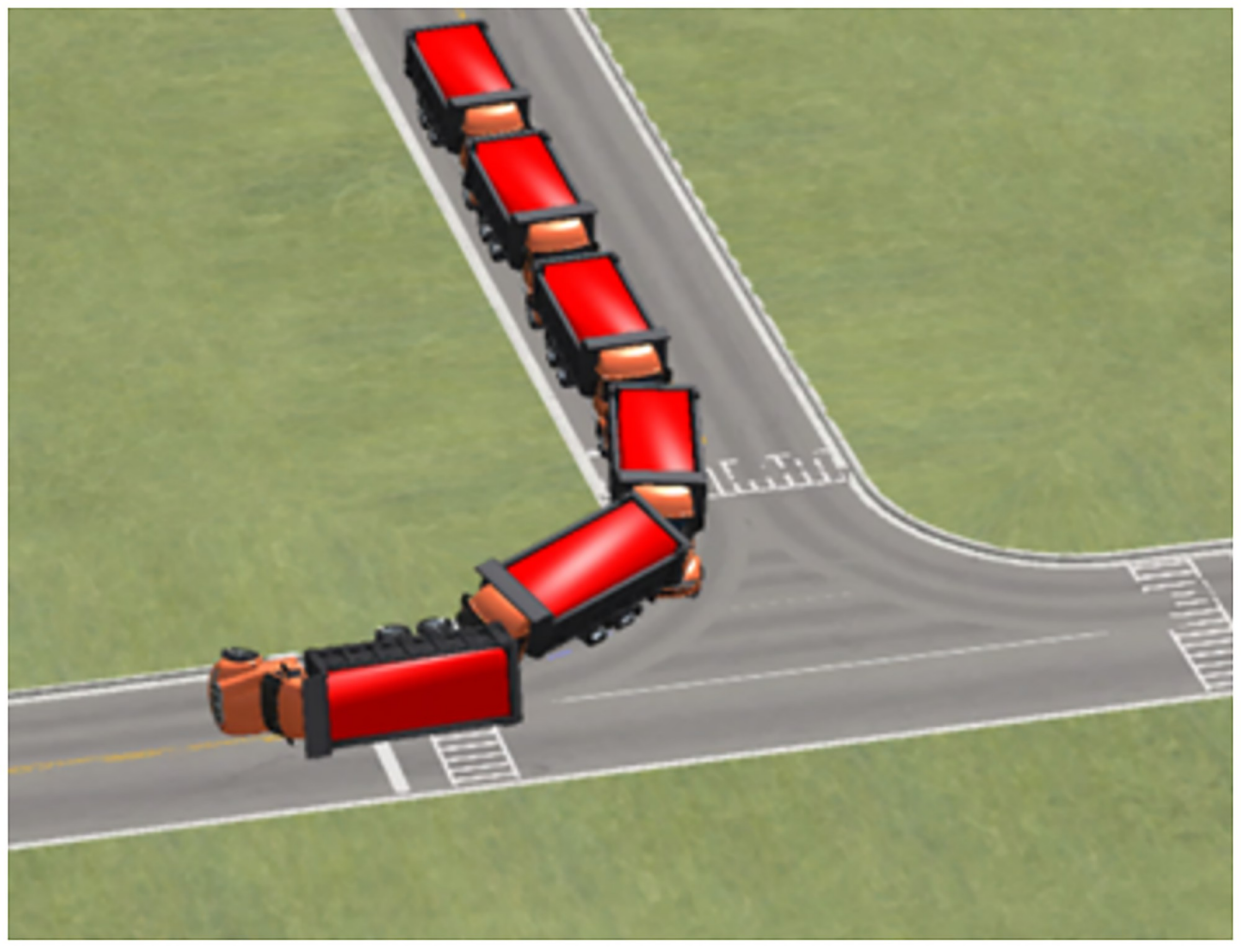
Vehicle rollover dynamic diagram.

Heavy trucks have the characteristics of a high center of gravity and high mass. Truck tipping can also occur due to lateral force when traveling fast on a curvy road (Fig 6).

Currently, the most used index for predicting vehicle stall condition is the *P*_*LTRd*_ [19] (lateral load transfer rate), which can accurately characterize vehicle stall instability. During a vehicle’s right turn, the load on the right-hand side of the wheel is transferred to the left-hand side. When *P*_*LTRd*_ = 1, the vertical force exerted by the vehicle’s right-hand wheel *F*_*R*2_ = 0, and the vehicle is in a rollover. When *P*_*LTRd*_ = 0.8, the vertical force of the vehicle’s right wheel *F*_*R*2_ > 0 but the risk of the vehicle rolling over is high [20]. *P*_*LTRd*_ is computed using Eq (2), expressed as:

$$\begin{array}{c} P_{LTR}=\frac{F_{L2}-F_{R2}}{F_{L2}+F_{R2}} \end{array}$$ (2)

Note: where *F*_*L*2_ is left-hand wheel side vertical force and *F*_*R*2_ the left-hand wheel side vertical force.

**Fig 7:**
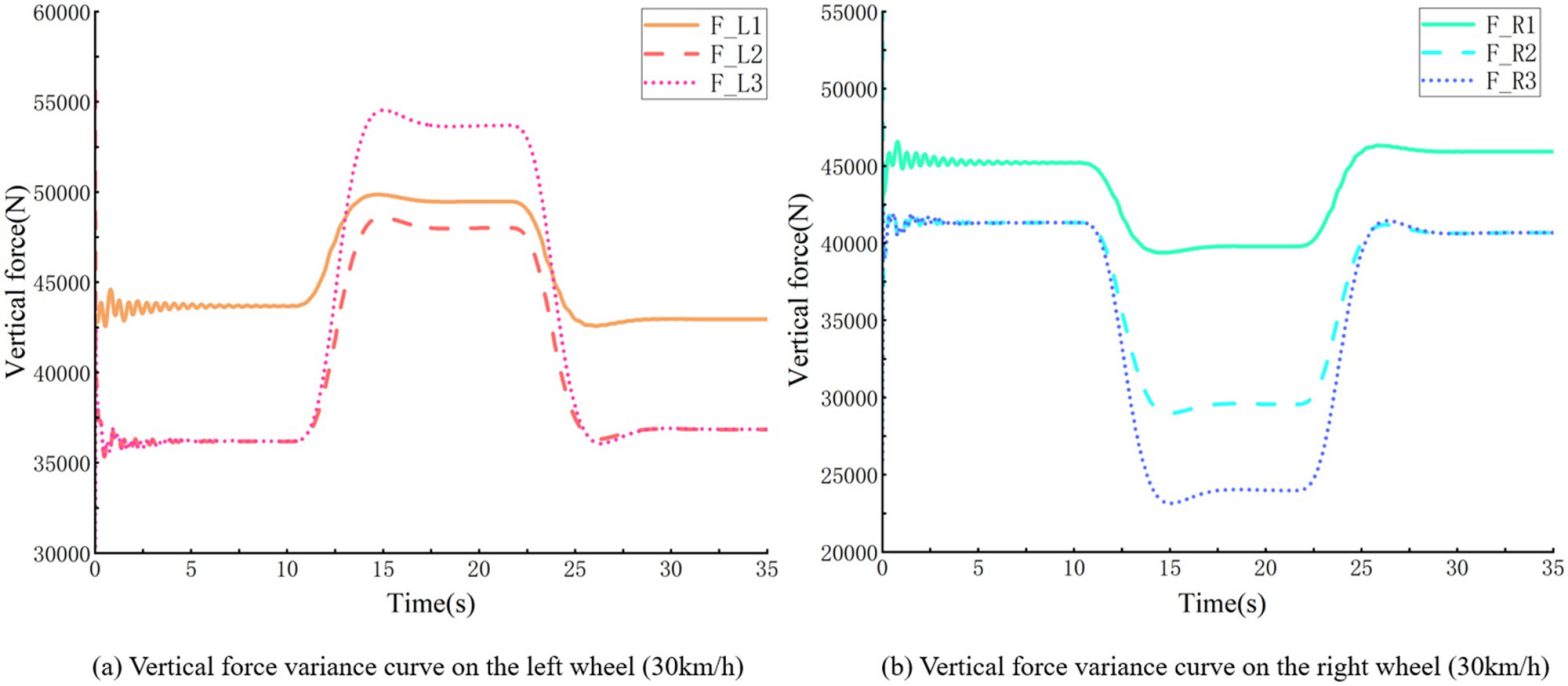
Vertical force curve of vehicle’s left and right wheels bending (30 *km*/*h*).

**Fig 8:**
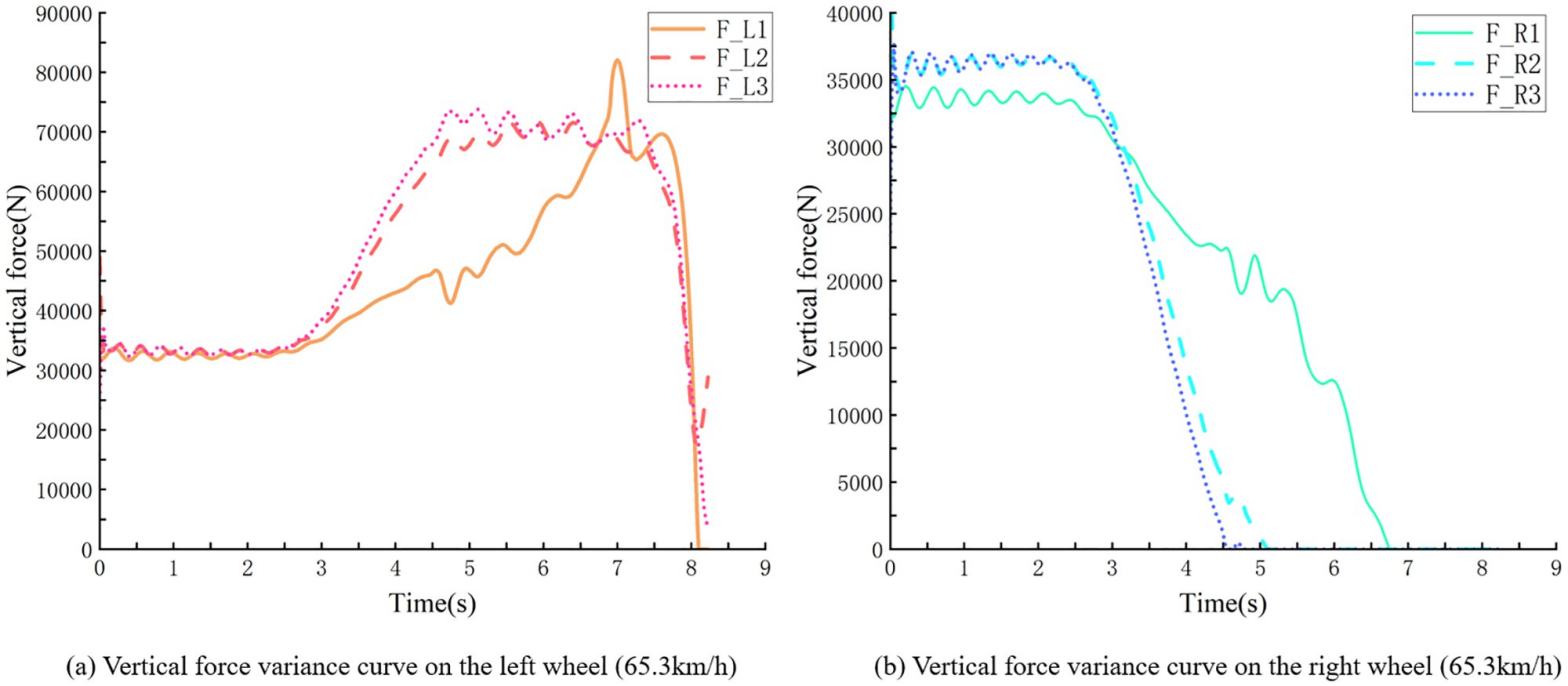
Vertical force curve of vehicle’s left and right wheels bending (65.3 *km*/*h*).

**Fig 9:**
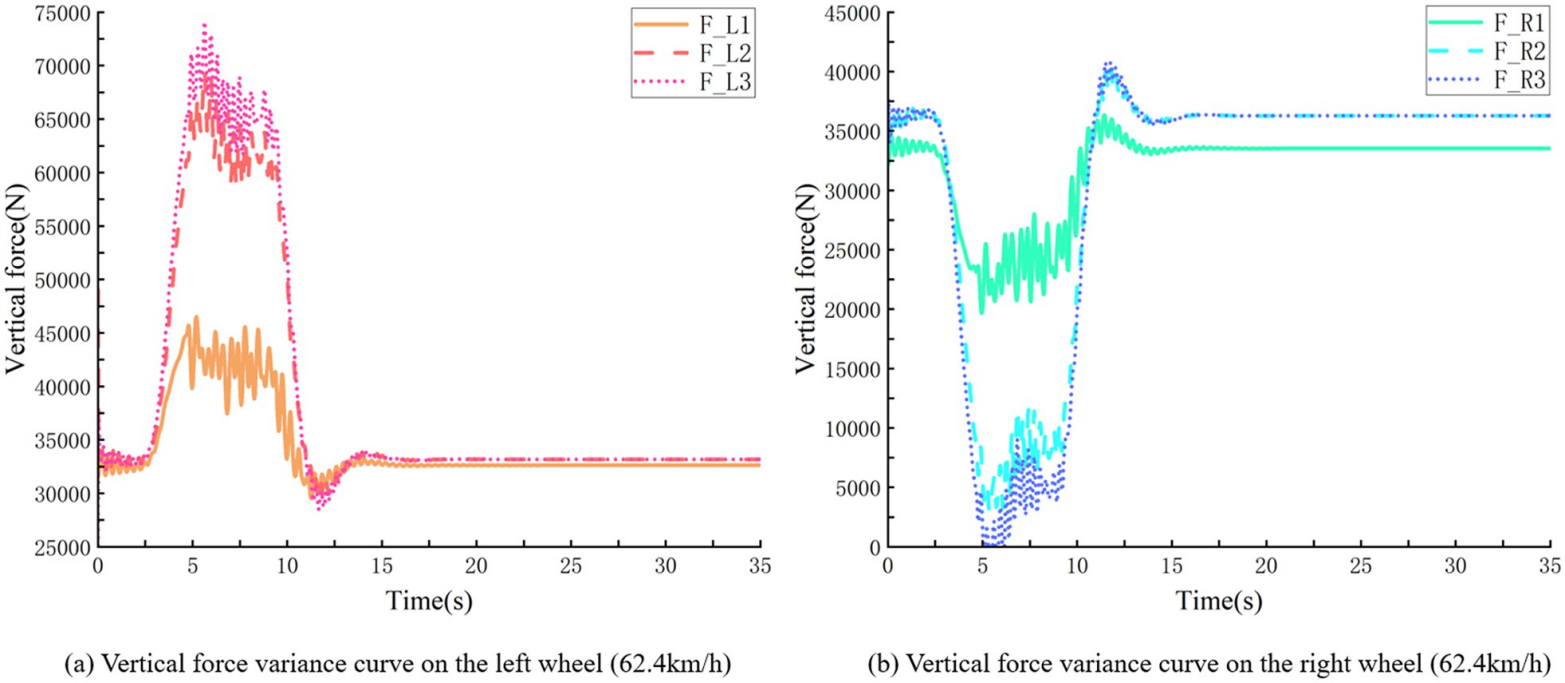
Vertical force curve of vehicle’s left and right wheels bending (62.4 *km*/*h*).

For example, within the 60 m radius of the right turn and with 0.7 condition adherence co-efficient, simulation truck descending at 30 km/h, and smooth turn of the vehicle, the minimum vertical force on the right wheel is greater than 0 N (Fig 7 (a) and 7(b)). Increasing the velocity to continue the simulation, with 65.3 km/h, at the corner, the vehicle experienced a rollover, and the vertical force of the right wheel rollover was constant at 0 N (Fig 8 (a) and 8(b)). Speed reduction continued to be simulated, with the vehicle having a straight wheel with a minimum vertical force close to 0 N at 62.4 km/h (Fig 9 (a) and 9(b)), and a significant tendency to roll to the side at the corners. Under these conditions, the vehicle’s velocity was used as the threshold for the vehicle’s unsteady velocity.

## Experiments in simulation

### Influence factors and sensitivity analysis

Due to truck characteristics, in the long descent into the driving process to the right T-junction, it was easy for the vehicle to exceed the recommended speed, progressing to instability and then skidding or rolling over [21]. To investigate the instability speed threshold of the truck turning right at long downhill T junctions, an analysis of influence factors and sensitivity was performed, and the common values of each factor in the real route selected for orthogonal experimentation. In these experiments, three levels of a four-factor orthogonal experiment were designed for investigation.

As the test instability velocity threshold did not involve the vehicle’s braking model, the non-braking closed loop driving model was chosen. That is, with the vehicle at a fixed speed in the curve, if the vehicle experienced a side slip or cartwheel, then the simulation was stopped; otherwise, the speed was increased and the simulation continued. The results of orthogonal experiments are shown in Table 2.

**Table 2:** Results of orthogonal experiment *L*_9_(3^4^) on factors affecting speed threshold of vehicle instability.

Influential factor	Attachment coefficient	Radius (m)	Superelevation	Vehicle overweight	Velocity threshold for instability (km/h)
1	1(0.2)	1(20)	1(0%)	1(0%)	19.9
2	1(0.2)	2(60)	2(2%)	2(20%)	39.2
3	1(0.2)	3(100)	3(4%)	3(50%)	49.8
4	2(0.5)	1(20)	2(2%)	3(50%)	25.8
5	2(0.5)	2(60)	3(4%)	1(0%)	62
6	2(0.5)	3(100)	1(0%)	2(20%)	67
7	3(0.7)	1(20)	3(4%)	2(20%)	32
8	3(0.7)	2(60)	1(0%)	3(50%)	44.7
9	3(0.7)	3(100)	2(2%)	1(0%)	73.6
i	36.300	25.900	43.867	51.833	
ii	51.600	48.633	46.200	46.067	
iii	50.100	63.467	47.933	40.100	
Extreme difference	15.300	37.567	4.066	11.733	

The experimental results showed that the turning radius had the greatest influence, which indicated that the radius had the greatest influence on the turning instability velocity threshold of the vehicle. Also, road adherence and vehicle overweight were secondary factors, and the superelevation of curved roads was a general factor of influence.

Orthogonal experiments were performed to obtain the instability speed threshold under different combinations of road parameters. To further investigate the influence of various factors on the unsteady speed thresholds of the vehicle, the control variate method was used to investigate the mechanism of influence of vehicle loading, excess turn height, and turn radius on the unstable speed threshold of the truck traveling in the right turn.

### Coefficient of adherence to roads

In this experiment, the actual vehicle data of a domestic brand of freight cars were used, and the maximum total mass of three axle freight cars set at 25 t after the full load of the freight cars, with reference to the provisions of Limits of dimensions, axle load and masses for motor vehicles, trailers and combination vehicles (GB 1589-2016) [22]. With reference to Identification for the speed of vehicle involved in road traffic accident (GB/T 33195-2016) [23], the minimum adhesion coefficient was 0.2, the maximum adhesion coefficient was 0.75, turning radius included 30, 60, and 100 m, and road superelevation was 2%. The simulation section consisted of downhill, curved, and straight sections.

**Fig 10:**
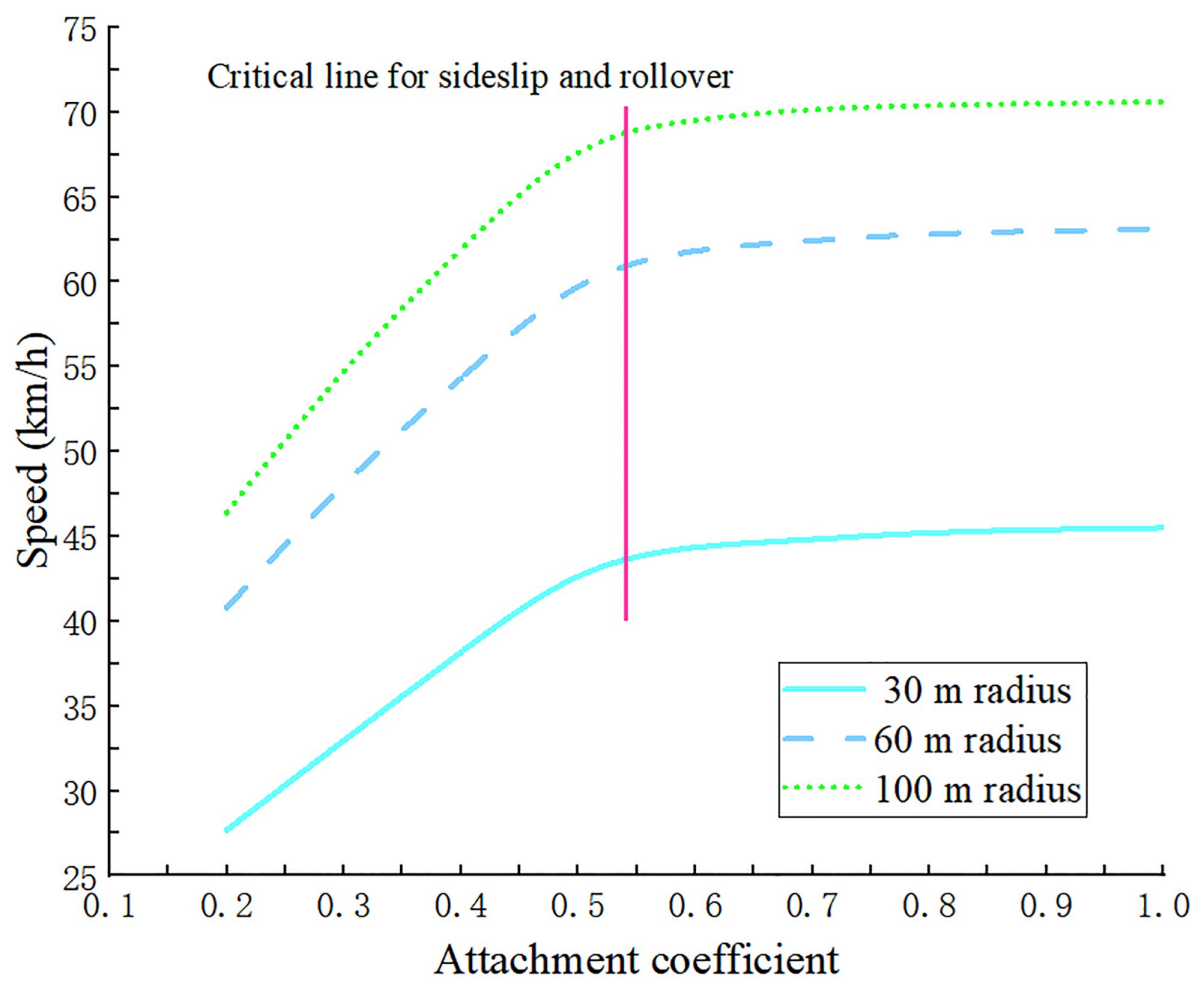
Curve of the instability speed of truck driving corresponding to different adhesion coefficient.

Simulation results showed that, based on the pattern of curve change, when the coefficient of adhesion was 0.5–0.6, all three curves had clear inflection points (Fig 10). The sensitivity of the instability velocity threshold was high prior to the inflection point and decreased with a decreased sticking coefficient. Beyond the inflection point, the instability speed threshold was less sensitive to the sticking coefficient and curve smoothness. The inflection points with radius of 30, 60, and 100 m appeared when the adhesion coefficient was 0.535. If the adhesion coefficient of the road was less than the point of inflection, truck adhesion of was low and it was difficult to balance the corresponding centrifugal force when the cornering speed was high. As a result, the truck had a sideslip and the load on the right side of wheel larger than 0 N, but the truck did not roll over. If the coefficient of adhesion to the road was larger than the abscissa of the inflection point, as the truck’s cornering speed increased and the load on the right side wheel transferred to the left side to 0 N, truck rollover occured, but the grip sufficient to balance the corresponding centrifugal force with no sideslip.

**Fig 11:**
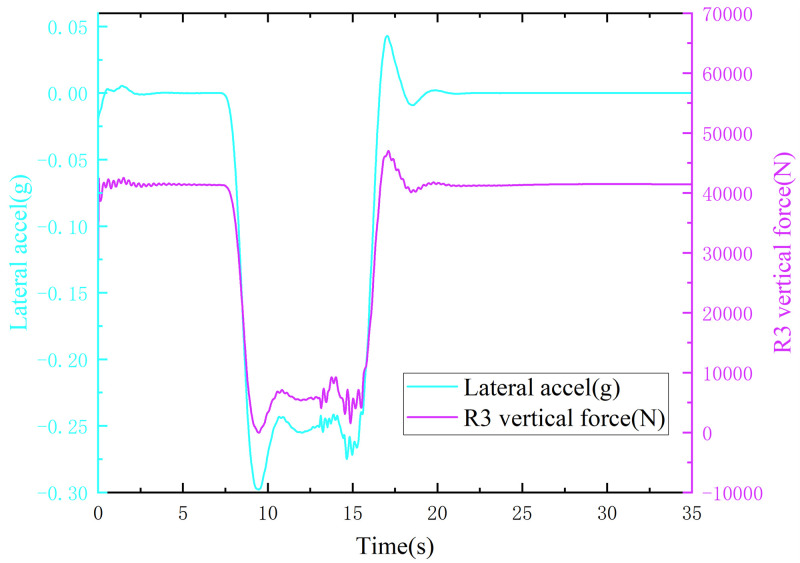
Lateral acceleration, right wheel vertical force curve (adhesion coefficient 0.30).

**Fig 12:**
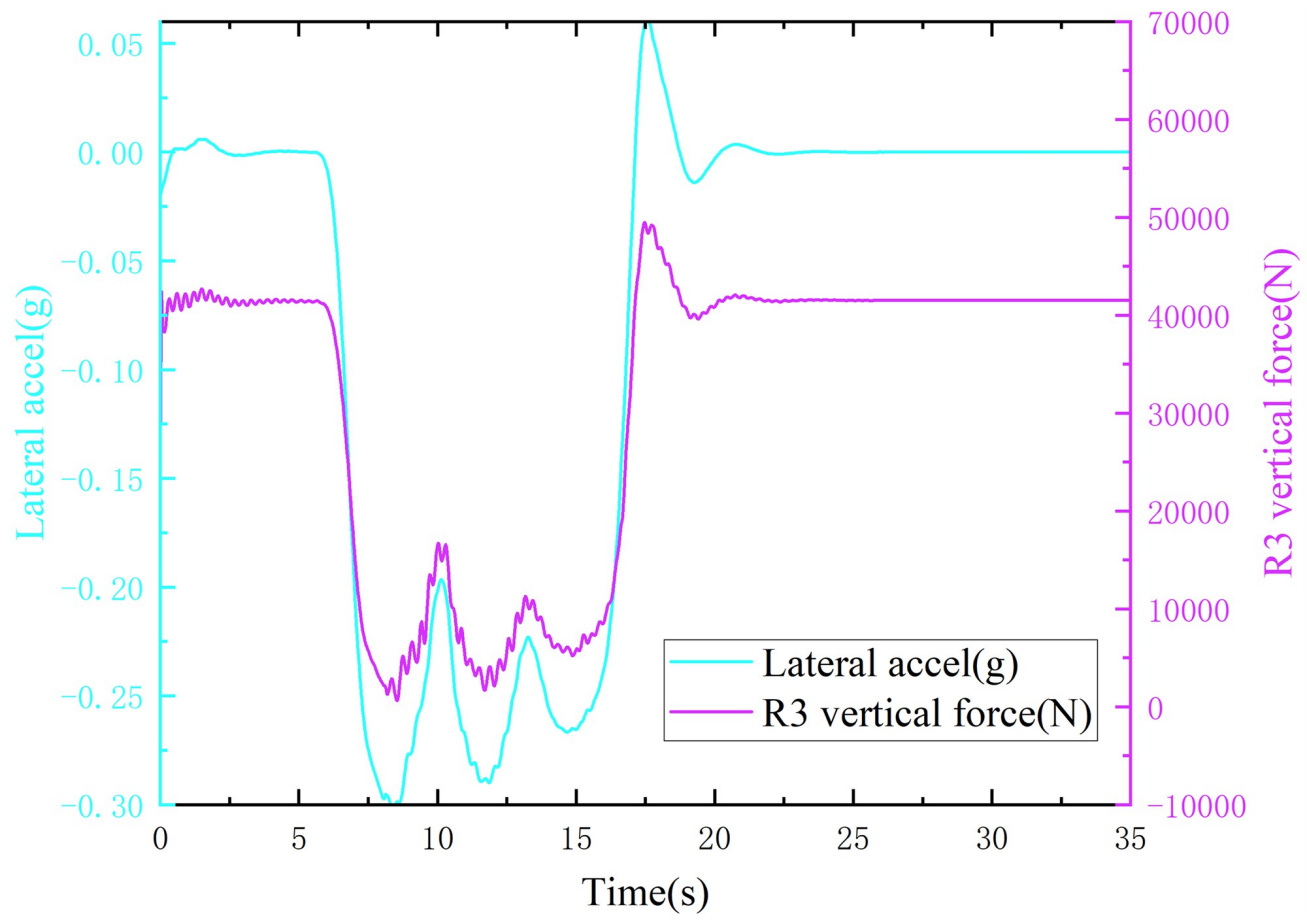
Lateral acceleration, right wheel vertical force curve (adhesion coefficient 0.70).

When the truck was driving on a road with a surface adhesion coefficient of 0.30, as the speed increased, the lateral acceleration of the vehicle was first greater than 0.3 g, while the right-hand wheel droop force of the third axle was greater than 0 N (Fig 11), indicating that the truck skidded without overturning. When the truck was driving on a curve with an adhesion coefficient of 0.70, the right-hand wheel droop force of the third axle of the wheel dropped to 0 N first, while the lateral acceleration of the vehicle was less than 0.3 g (Fig 12), indicating that the truck rolled over without skidding.

### Turning radius

The radius of curvature of the road was 20–100 m, according to the Code of Design for Highway Routes, and the road had a superelevation of 2%. All other conditions remain unchanged, and the pavement had high, medium, and low pavement adhesion coefficients of 0.7, 0.5, and 0.2, respectively.

**Fig 13:**
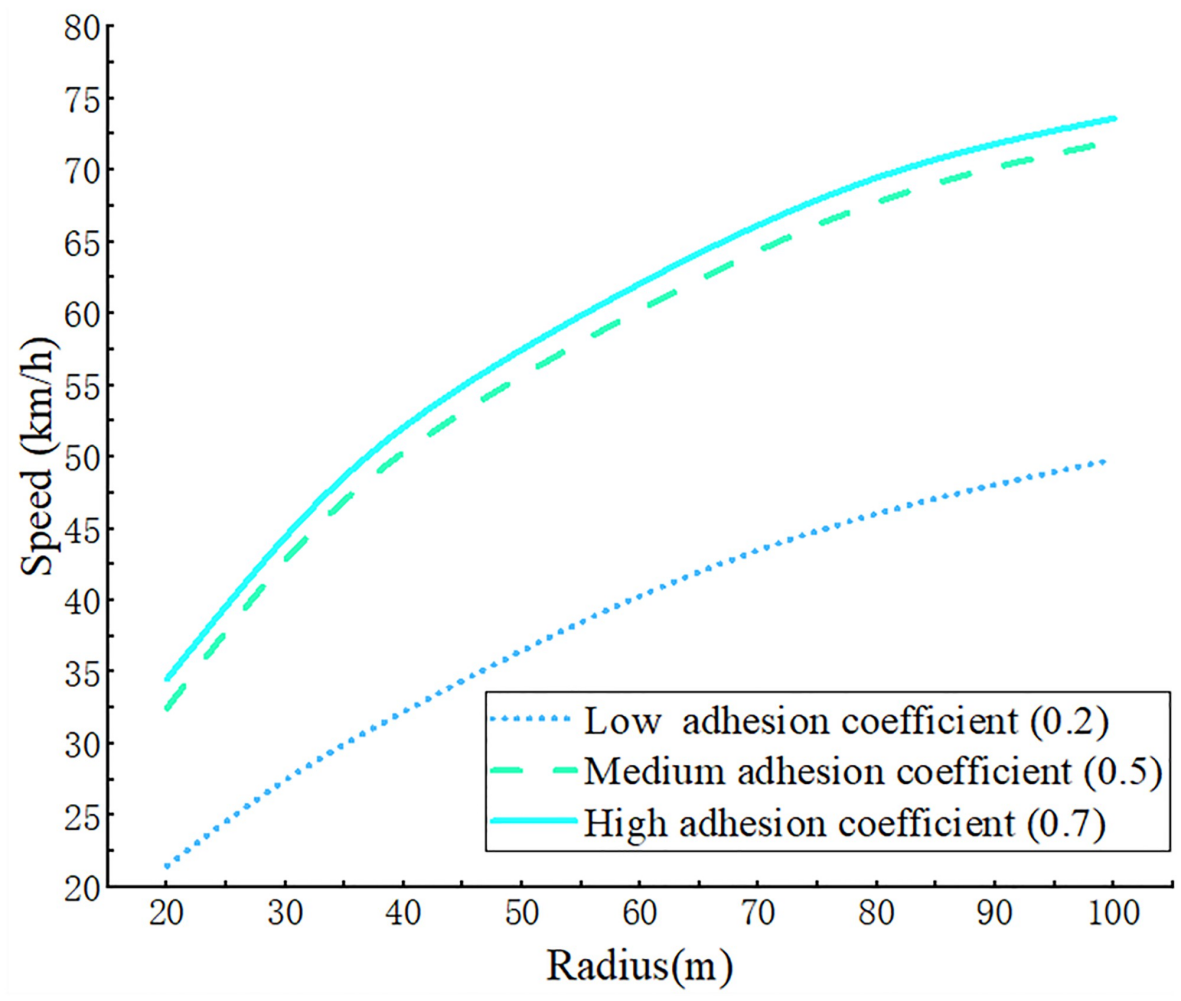
The curve of vehicle driving instability speed threshold corresponding to different road turning radius.

The results of simulation experiments with various combinations of parameters shown that, as the turning radius became smaller, the instability velocity threshold also decreased, and the curve changed more slowly as the turning radius increased (Fig 13). Also, at the same turning radius, the threshold for road instability velocity was high when the coefficient of adhesion was large. For example, when the turning radius was 80 m, the instability velocity threshold for full-load trucks was 70.2 km/h on a road with the coefficient of adhesion of 0.7 and a threshold at 68.5 km/h on a road with the coefficient of adhesion of 0.5. If the turning radius had the minimum value of 20 m, then the speed of full load trucks on roads with high, medium, and low adhesion coefficients was at a lower value, being 34.5, 32.5 and 21.5 km/h, respectively.

### Superelevation of the curve

**Fig 14:**
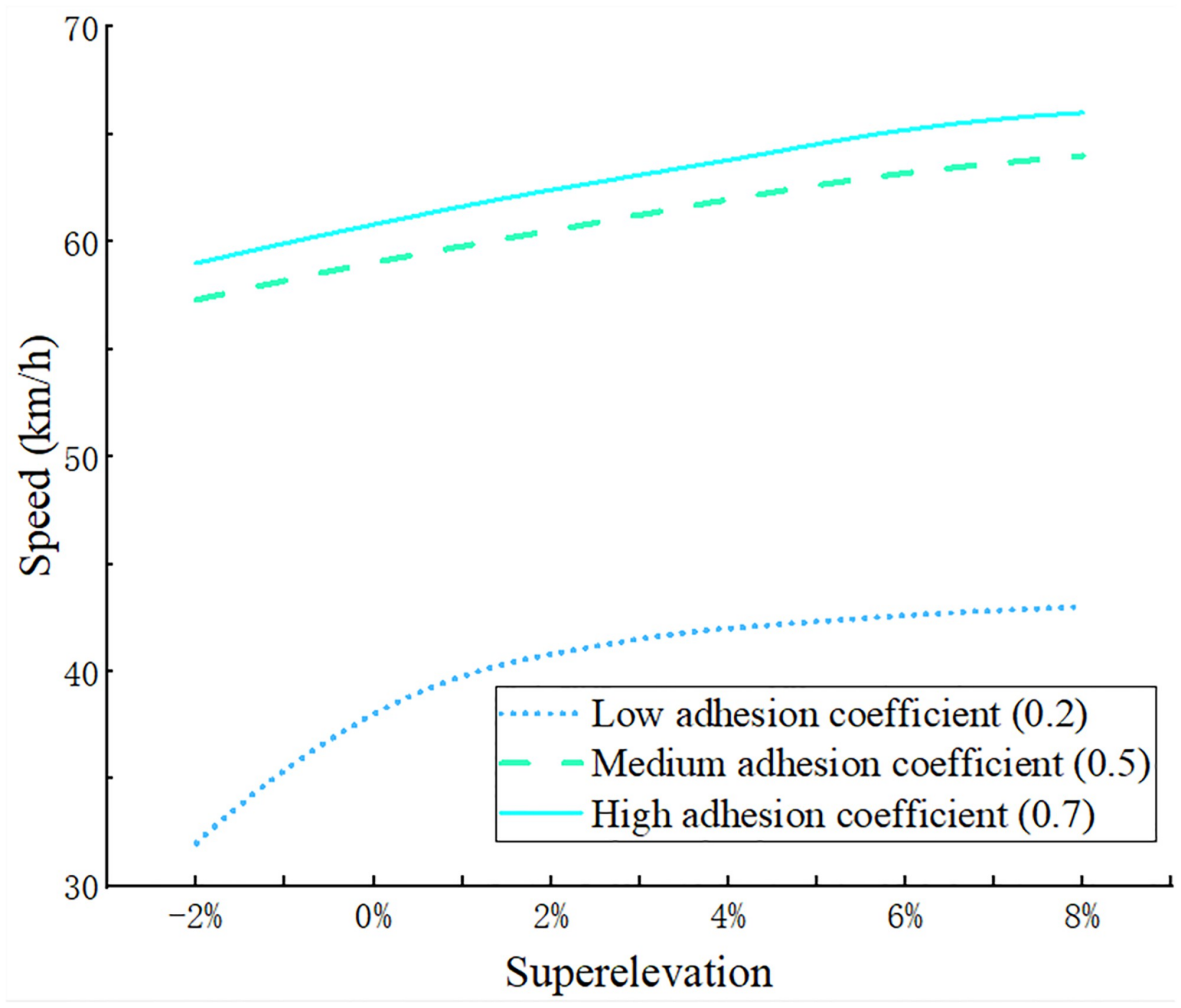
The curve of the instability speed threshold of truck driving corresponding to different road superelevation.

In the case of not changing vehicle conditions, three road adhesion coefficients were set and the road radius was set at 60 m, with other parameters not changed. Referring to the Code for the Design of Highway Routes for setting the superelevation value of the road curve to 0–8% and the reverse superelevation (road design or construction defects) considered to set the cross-slope of the curve to be -2%. The simulation results are shown in Fig 14.

The instability speed threshold of trucks running on high and medium adhesion coefficient roads was higher than that on low adhesion coefficient roads and the difference large. Driving on a road with a low adhesion coefficient created a high sensitivity to the instability speed threshold of the truck and a sharply changing curve. However, when the road superelevation was >4%, the curve effect decreased, with friction being small at this point and the truck being mostly balanced by centripetal and centrifugal forces. As the superelevation of the road became smaller, centripetal force diminished, and the right side wheel load increased toward the left side. If the lateral glide threshold sensitivity was low, the curve effect was less. Furthermore, in practice, due to design and construction flaws, this can occur when road superelevation is negative, e.g., when the adhesion coefficient was 0.2, the curvature slope was -2%, and instability speed threshold was 32 km/h, whereas the slope threshold was 2%, creating a threshold of 40.8 km/h.

### Truckload

**Fig 15:**
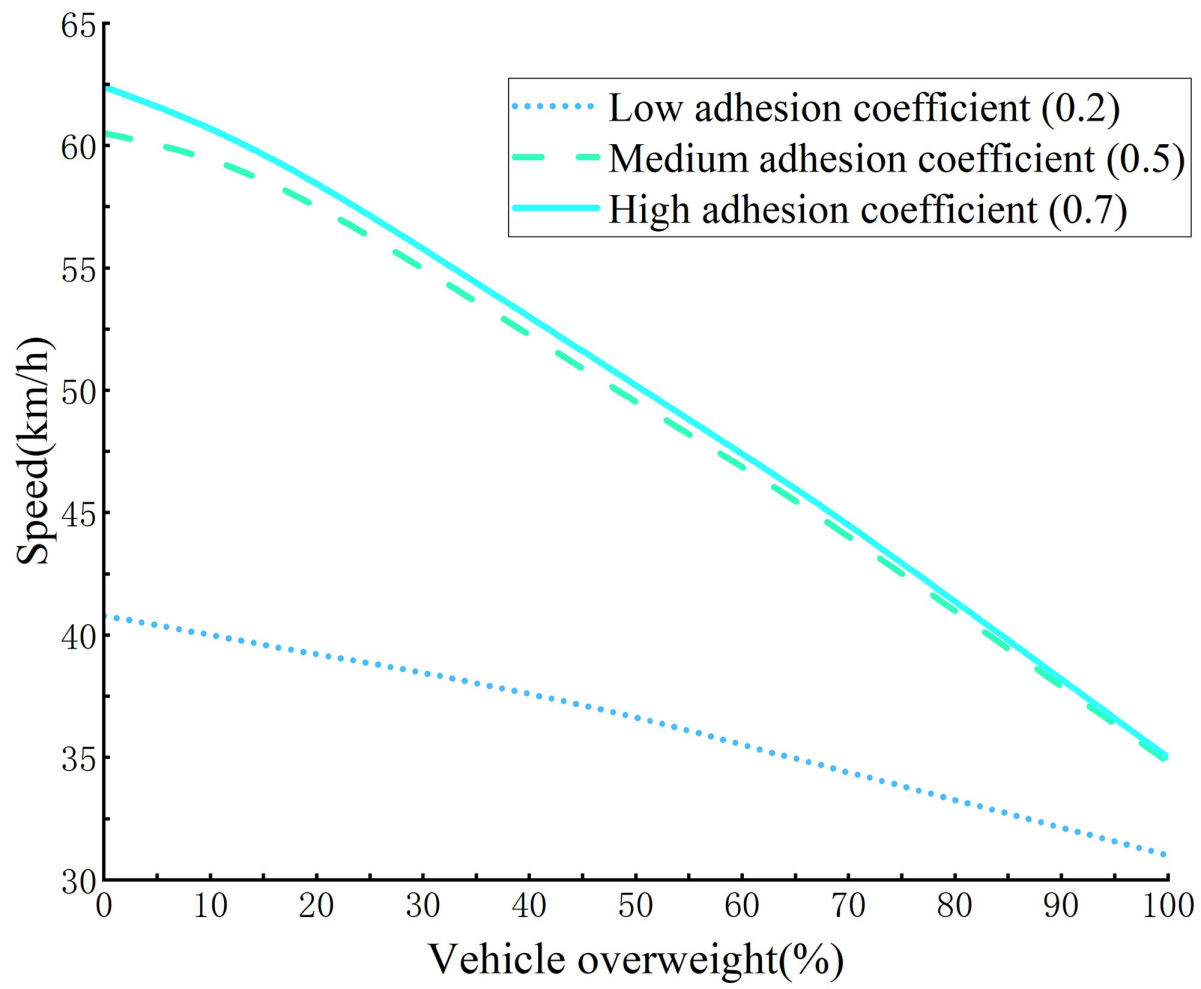
Curve of the instability speed threshold of truck driving corresponding to different loads.

According to the Limits of Overall Dimensions, Axle Load, and Mass of Automobiles, Trailers, and Motor Trains, the maximum total mass of three freight cars with axles is 25 t. Here values were set to be 0-100% of the weight of an overweight truck. Other conditions remained constant, including adhesion coefficient roads were set to high, medium, and low, turning radius at 60 m, and road superelevation at 2%. The results of the simulation after the truck was overloaded show that the overall centroid of the vehicle became higher and its stability decreased, with the vehicle instability threshold velocity decreasing with increased vehicle overload weight, which was negatively correlated and highly variable (Fig 15).

In addition, the observed pattern of curve change showed a curve sensitivity to truck weight which became more significant after the overweight vehicle reaches 30% (Fig 15). The instability velocity threshold was at an extremely low velocity on high, medium, and low adhesion coefficient routes. Thus, the instability velocity threshold was <35 km/h when the coefficient of adhesion was 0.7 and the truck 100% overweight (the truck total mass was 50t). When the truck was not overloaded, the instability velocity threshold was 62.4 km/h.

## Analysis of accident causes and countermeasures

### Analysis of accident causes

**Fig 16:**
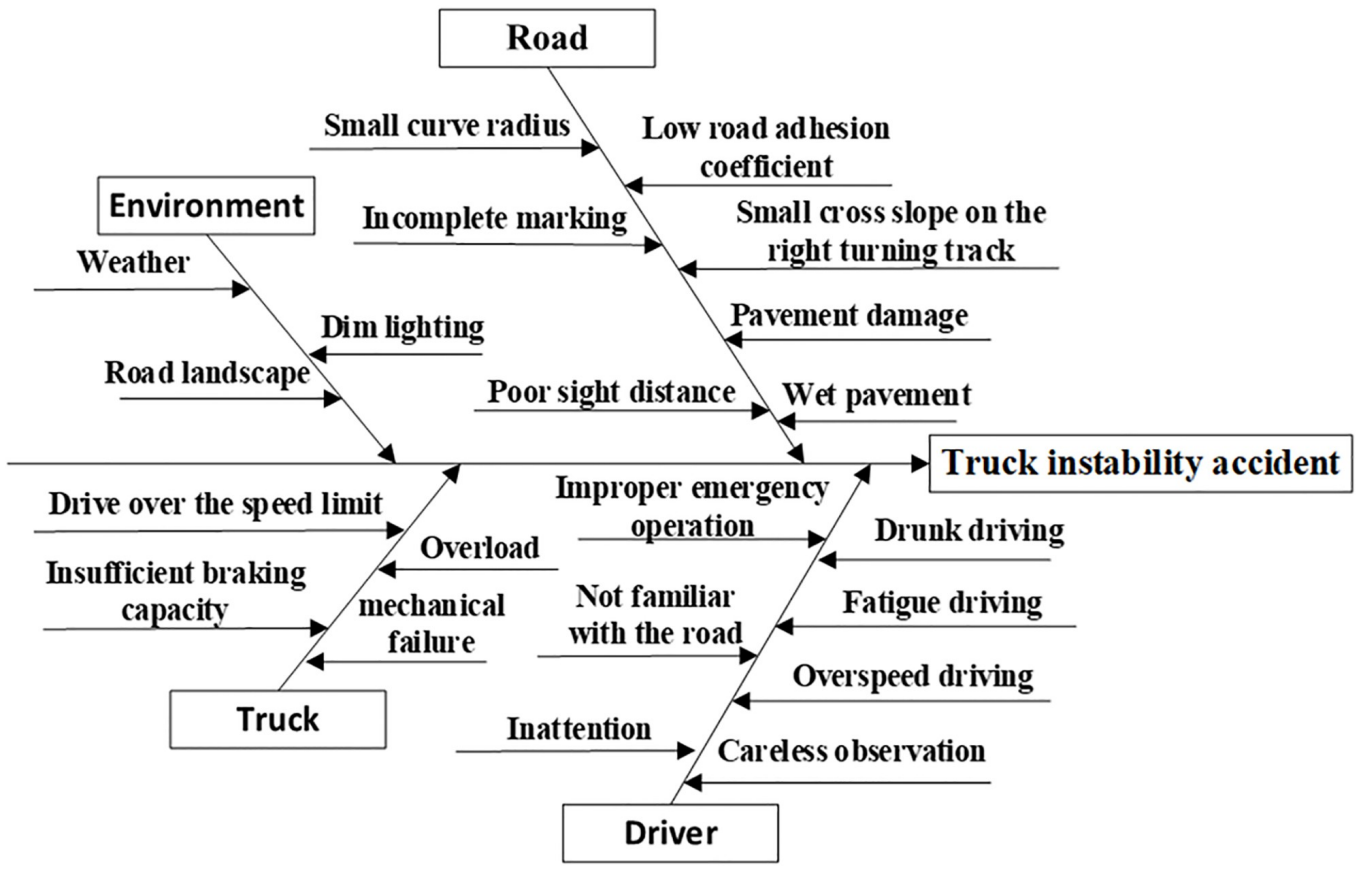
Fishbone diagram of cause mechanism of truck right turning instability accident at T-shaped intersection on long downhill.

At the T-intersection at the bottom of the long downhill roadway, the conditions for trucks to safely turn right were relatively harsh, requiring people, vehicles, and roads to be in good engineering condition. In general, there might be a variety of adverse factors (conditions) that cause a truck to lose stability when turning right at a long downhill T-junction (Fig 16). Judging from many of the accident cases that have occurred, right-turn crashes occur frequently at T-intersections on long downgrades. The six factors responsible for most of them are as follows:

1. The right turn lane was not specifically designed, and the design radius of the vehicle’s right turn trajectory curve small. Previous research has shown that the turning radius is the most important factor affecting the threshold of the truck’s turning instability speed, with the smaller the turning radius, the lower the instability speed threshold. Road engineering standards specify minimum turning radii at different design speeds. However, most of the two roads that intersect at the actual intersection were built in different periods. When the intersection was formed, due to terrain, features, land rights, road rights, and other factors, the designer or construction party did not have sufficient knowledge of the right-turn lane. As a result, the designer often did not establish a right-turn lane consistent with the right-turn trajectory curve, such that the actual radius of the right turn was small, which did not meet the demand for the right turn radius at the bottom of the long downhill slope. There are also some T-junctions where, although the right turning radius is sufficient, due to the non-standard pavement markings, poor visibility conditions in the triangle area, and roadside traffic management, the actual track curve radius of vehicles turning right are objectively limited. A right T-intersection turn curve example here has a design radius of 30 m, which corresponded to a minimum design velocity of 30 km/h. However, the effective real radius was 21.2 m, which cannot be traversed at 30 km/h (Fig 2).
2. There are defects in the construction of the road frontage at the intersection, and the cross-slope of the road on the right-turn lane low. During the design and construction of the road, the cross slope of the crown with high, middle, and low sides are fixed in the straight section of the road to facilitate transverse drainage of the roadway. When a downhill road intersects a straight road, it is necessary to relate the transverse and longitudinal slopes of the downhill road to the transverse and longitudinal slopes of the road being traversed (i.e., elevation design). As there are many controlling factors for the design and construction of the facade of the intersection and the requirements for the construction process are high, the facade of some T-intersections with long, downhill bottoms is not strictly built to specification, leading to an insufficient cross-slope of the road surface on the right-turn lane and even an adverse slope. As an example, for a right-turn bend with a radius of 60 m and adhesion coefficient of 0.7, the instability threshold on a 2% superelevation pavement was 62.4 km/h, while the instability threshold on the -2% superelevation pavement was 59 km/h.
3. At the intersection, road maintenance lags behind, and there are some potholes or other extraneous issues on the right-turn road. As the truck turns to the right, the bearing weight of the left and right wheels varies widely. The road surface of the left wheel track belt is more easily damaged than the road surface of the right wheel, such that the road surface of the track belt on the left wheel is subject to rutting, sinking, bumping, and other illnesses. Also, some vehicle loads, such as sand and gravel, are also unstable and easily dispersed on the road during cornering. On one hand, potholes or foreign issues on the road interfere with the driver’s emergency operation while. On the other hand, it is easy to break the stability of a vehicle that is running at the edge of the balance boundary, resulting in the sudden instability of the vehicle.
4. Traffic signs are missing or set incorrectly. Before turning right at the T-intersection at the bottom of the long downslope, to control the speed, the vehicle must obtain safe driving speed information from traffic signals; Vehicle drivers must also obtain information about the shape and direction of the intersection in front of them before arriving at the intersection, to help them select the correct driving route. At T-intersections at the bottom of long downhill grades, where a high number of accidents occur, there are often few or no speed limit signs, guide signs, or intersection warning signs; some of which were set at incorrect positions and failed to convey the correct information to the driver in the correct position.
5. Vehicle speeding or overloading when turning right. Previous research has shown that over speeding and overloading both have a significant negative impact on the instability velocity threshold of right-turning vehicles. For example, the threshold value of an unsteady velocity of a non-overloaded vehicle (25 t) on a curve with a radius of 60 m and an adhesion coefficient of 0.7 was 62.4 km/h. However, the instability velocity threshold for a 100% overweight vehicles was reduced to 35 km/h. In contrast, truck over speeding and overloading are often the most direct reasons for worsening the severity of the consequences of a crash. Truck instability accidents due to overspeeding and overloading are driven primarily by interests, as the drivers are unfamiliar with the vehicle and road conditions and do not carefully control the vehicles’ right-hand turn after a long descent.
6. If the brake ability on the vehicle decreases or the driver’s emergency operation is inadequate after driving down a long hill. Frequent braking is often required to control the speed of trucks when driving on long downhill sections, which results in an increase in vehicle brake temperature and decreased heat. As a result, brake effectiveness worsens, causing failure of the vehicle to decelerate to a safe speed prior to entering the curve; the driver of the vehicle might also have improper emergency operations, which eventually result in accidents.

### Countermeasures

1. Fine design and construction of right-turn road junctions. The long, downhill T-junction has a special structure that is a combination of a ramp and a curve. This might reduce the gradient of the access section of the system and establish scientifically and normatively the vertical structure of the domain of intersection to prevent the sudden change in horizontal, vertical, and horizontal linear parameters on the right-turn trajectory. However, the turn radius can be increased if conditions allow, and full and continuous right-turn lanes can be established. In addition, obstructions in the right turn intervisibility triangle area should be removed as far as possible to ensure adequate sight distance.
2. A variety of traffic signs and step-by-step speed limit signs must be installed in a standardized manner. Guide signs (or warning signs at intersections) that clearly reflect the shape and direction of the intersection must be placed in advance before the T junction is approached. There are speed limit signs in front of the right-turn lane. In long downhill sections, if the speed limit is high before the turn, a graded speed limit sign might be placed before approaching the T-intersection to avoid an excessive speed limit range. Also, in the direction facing long descending traffic, linear guidance signs might also be placed to remind the driver to turn to the right or left.
3. Road traffic markings shall be set in a standardized way, and speed humps shall be set if necessary. The T junction can be canalized. Diverting marks (or diversion islands), guiding arrows, lane edge lines and boundaries, and lateral oscillation deceleration marks can be used to provide clear right-turn right-of-way information for right-turning vehicles, to drive with caution, and to avoid accidents.
4. Road maintenance needs to be strengthened to ensure that signage and markings are effective, and that the road surface is free of foreign issues. Worn marks and branding must be replaced or repaired in due course. Foreign matter must be kept off the pavement in a timely manner. If necessary, the pavement can be repaved.

## Results and discussion

1. Trucksim simulation software was used to establish a simulation vehicle system. Starting from the control variate method, simulation experiments are performed for different bending conditions, and the instability velocity threshold of flexural freight cars discussed. In this study, influential factors were analyzed using orthogonal experiments and the results showed that four influencing factors were interrelated and jointly affected the buckling(instability) speed threshold of the vehicle as it passes through a corner. Of the four influencing factors, the turning radius had the greatest influence on the vehicle threshold value of the buckling speed as it passed a corner, with the secondary factors being the road adhesion coefficient and vehicle load; and road superelevation a general factor.
2. It can be seen from the results of the single factor experiment that, with increasing turn radius, superelevation, and road adhesion coefficients, the threshold value of vehicle buckling speed gradually increased; with increased in vehicle load, the threshold of vehicle bending instability speed decreased gradually. As the truck’s speed increased, when driving on a road with a low coefficient of adhesion, at first, the truck skids and then rolls over. When the truck is traveling on a road with a medium or high coefficient of adhesion, it traverses right over it.
3. This study was based as Trucksim simulation experiments and analyzed the causes of sideslip and rollover instability accidents of freight trucks at a long downhill T junction and advances the corresponding crash safety countermeasures, which would be useful for improving the traffic safety of this special road section.

## Supporting information

S1 Dataset (DOCX)

S1 File (DOCX)
