# Supplementary material for: Study of instability mechanisms of trucks turning right at long downhill T-junctions based on Trucksim simulation
Source: PLoS One. 2023 Mar 8;18(3):e0282779. doi: 10.1371/journal.pone.0282779 (PMC9994748; doi:10.1371/journal.pone.0282779)
Supplement: S1 Dataset — (DOCX) [file pone.0282779.s001.docx]

The curve of vehicle driving instability speed threshold corresponding to different road turning radius.

| Radius(m) | Low adhesion coefficient (0.2) | Medium adhesion coefficient (0.5) | High adhesion coefficient (0.7) |
| --- | --- | --- | --- |
| 20 | 21.5 | 32.5 | 34.5 |
| 30 | 27.7 | 43.35 | 44.8 |
| 40 | 32.3 | 51 | 52.8 |
| 60 | 40.8 | 60.5 | 62.4 |
| 80 | 46.4 | 68.5 | 70.2 |
| 100 | 49.8 | 72 | 73.6 |

The curve of the instability speed threshold of truck driving corresponding to different road superelevation.

| Superelevation | Low adhesion coefficient (0.2) | Medium adhesion coefficient (0.5) | High adhesion coefficient (0.7) |
| --- | --- | --- | --- |
| -0.02 | 32 | 57.3 | 59 |
| 0 | 38 | 59.1 | 60.8 |
| 0.02 | 40.8 | 60.5 | 62.4 |
| 0.04 | 42 | 62 | 63.8 |
| 0.06 | 42.6 | 63.3 | 65.2 |
| 0.08 | 43 | 64 | 66 |

Curve of the instability speed threshold of truck driving corresponding to different loads.

| Vehicle overweight (%) | Low adhesion coefficient (0.2) | Medium adhesion coefficient (0.5) | High adhesion coefficient (0.7) |
| --- | --- | --- | --- |
| 0 | 40.8 | 60.5 | 62.4 |
| 10 | 40 | 59.6 | 60.8 |
| 20 | 39.2 | 57.6 | 58.5 |
| 30 | 38.5 | 55 | 55.8 |
| 50 | 36.7 | 49.5 | 50.2 |
| 60 | 35.5 | 46.9 | 47.4 |
| 70 | 34.4 | 44.1 | 44.6 |
| 100 | 31 | 34.8 | 35 |
