# Supplementary material for: Study of instability mechanisms of trucks turning right at long downhill T-junctions based on Trucksim simulation
Source: PLoS One. 2023 Mar 8;18(3):e0282779. doi: 10.1371/journal.pone.0282779 (PMC9994748; doi:10.1371/journal.pone.0282779)
Supplement: S1 File — (DOCX) [file pone.0282779.s002.docx]

PARSFILE

#FullDataName TruckSim Run Control`Baseline #1`* * Quick Start Guide Example

#VEHICLECODE S_SS

symbol_push <<unit>> 0

symbol_push <<axle>> 0

symbol_push <<s>> 0

symbol_push <<o>> 0

symbol_push <<id_run>> Run_fbbb29b5-35c6-40f2-96a9-e83cc7f81ac7

OPT_ALL_WRITE 0

IOBJECT 0

OPT_INT_METHOD 0

OPT_VS_FILETYPE 2

PARSFILE Animator\Cameras\Camera_1cda02f1-83b2-420a-a133-a4725146fd4e.par

#BlueLink0 Animator: Camera Setup`25 deg. Azimuth, Veh. Ref.` Vehicle Reference` , Animator camera`Camera_1cda02f1-83b2-420a-a133-a4725146fd4e

#CheckBox0 1

#CheckBox1 0

#CheckBox2 0

#CheckBox3 0

#CheckBox4 1

#CheckBox5 0

#CheckBox6 0

#CheckBox7 0

#CheckBox8 0

#CheckBox9 1

#CheckBox10 0

#CheckBox11 0

#RingCtrl0 4

#RingCtrl1 0

#RingCtrl2 3

#RingCtrl3 0

#RingCtrl6 2

#RingCtrl7 -1

*RUN_COLOR 1 0.4 0.2

SET_RUN_COLOR 1 0.4 0.2

#RingCtrl4 off

RT_WINDOW_CLOSE off

#RingCtrl5 0

LIVE_SERVER_MAX_CONNECTIONS 0

PARSFILE Vehicles\Lead_3a\Lead3a_58294193-48b0-4db2-8d8e-da91df7077ff.par

#BlueLink2 Vehicle: Lead Unit with 3 Axles`3A Dump Truck #2` 3A Dump Truck` , Math Model`Lead3a_58294193-48b0-4db2-8d8e-da91df7077ff

PARSFILE Procedures\Proc_71be3a85-2564-4531-a2bd-13d1bb3b32cc.par

#BlueLink28 Procedures`Intersection, 3-Way with Curbs (Straight Trucks) #1` Road Networks` , Procedure`Proc_71be3a85-2564-4531-a2bd-13d1bb3b32cc

*TSTOP 1

PARSFILE Plot\Setup\Plot_0616a266-66af-4221-a68a-ad24f1a280e3.par

#BlueLink15 Plot: Setup`Vehicle Sideslip Angle vs. Time` Vehicle Motion` , Plot`Plot_0616a266-66af-4221-a68a-ad24f1a280e3

*IMAGE_TYPE PASS_THROUGH

*IMAGE_PASS_THROUGH Animator\Vehicles\AniVeh_5e99b800-fddc-4e1b-a23d-d8a55608d47a.png

OPT_ALL_WRITE 1

WRITE_SENSOR_DETECT

Title Baseline #1 <* * Quick Start Guide Example>

CATEGORY * * Quick Start Guide Example

DATASET_TITLE Baseline #1

#RingCtrl8 1

*NUM_CACHED_RUNS 1

#RingCtrl9

#EMBEDDED_NOTES

#This is a baseline example used in the TruckSim Demo Tutorial (see Help > Guides and Tutorials). It is similar to the example used in the TruckSim Quick Start Guide.

#

#Note: This example has the box checked to "Write all outputs." This simplifies the use of this run as a reference for comparisons with other runs that might have different plot settings.

#END_EMBEDDED_NOTES

LOG_ENTRY Used Dataset: TruckSim Run Control; { * * Quick Start Guide Example } Baseline #1

#Library : TruckSim Run Control

#DataSet : Baseline #1

#Category: * * Quick Start Guide Example

#FileID : Run_fbbb29b5-35c6-40f2-96a9-e83cc7f81ac7

#Product : TruckSim 2019.0

#VehCode Run

END
